# Effects of Recent Air Pollution Exposure on Children’s Neurocognitive Functions and the Possible Link with Endothelial Function

**DOI:** 10.3390/ijerph23081070

**Published:** 2026-08-18

**Authors:** Hanne Hendrickx, Annelies Van Eyck, Kristien Wouters, Julie Degraeve, Kevin Lamote, Roeland Samson, Stijn Verhulst

**Affiliations:** 1 Laboratory of Experimental Medicine and Pediatrics (LEMP), University of Antwerp, Universiteitsplein 1, Wilrijk, 2610 Antwerp, Belgium; 2 Biobased Sustainability Engineering (SUSTAIN), Department of Bioscience Engineering, University of Antwerp, Groenenborgerlaan 171, 2020 Antwerp, Belgium; 3 Department of Paediatrics, Antwerp University Hospital, Drie Eikenstraat 655, 2650 Edegem, Belgium; 4 Faculty of Medicine and Health Sciences, University of Antwerp, Universiteitsplein 1, Wilrijk, 2610 Antwerp, Belgium; 5 Clinical Trial Centre (CTC), CRC Antwerp, Antwerp University Hospital, Drie Eikenstraat 655, 2650 Edegem, Belgium

**Keywords:** air pollution, pediatrics, endothelial function, neurocognition, schoolchildren

## Abstract

**Highlights:**

**Public health relevance—How does this work relate to a public health issue?**

- Air pollution in school environments may affect children’s cardiovascular and neurocognitive health.
- Children are exposed to particulate matter during a substantial part of their daily activities at school.

**Public health significance—Why is this work of significance to public health?**

- Increased particulate matter exposure was associated with reduced microvascular dilatation in healthy schoolchildren.
- School environment characteristics, including open-air schooling, may influence children’s exposure and related health outcomes.

**Public health implications—What are the key implications or messages for practitioners, policy makers and/or researchers in public health?**

- Improving air quality in and around schools may support healthier learning environments.
- Larger longitudinal studies with more accurate exposure assessment are needed to clarify the effects of school-related air pollution on child development and health.

**Abstract:**

Air pollution poses a major health risk, particularly in children, who spend considerable time in school. We monitored air pollution in school environments and investigated its impact on cardiovascular and neurocognitive outcomes. Seven elementary schools in Antwerp, Belgium, including one open-air school, participated in a longitudinal study. Indoor and outdoor particulate matter (PM) and outdoor NO_2_ concentrations were measured. Endothelial function and attentional outcomes were assessed repeatedly (*n* = 3) in 138 healthy children (mean age of 10.3 ± 0.5 years, 55.8% male). Mixed effect models were used to investigate the association between PM and NO_2_ exposure and endothelial and attentional outcomes. This study considered median PM concentrations during the 4 h of the study visits (direct exposure), those during the 24 h (acute exposure) and 1 week preceding the study visits (recent exposure), and monthly NO_2_ concentrations. Models were adjusted for sex, age, BMI-z, and visit. Increased direct (4 h) indoor PM exposure was associated with reduced microvascular dilatation, while selective and sustained attention improved with direct and recent PM exposure, respectively. No associations were found with NO_2_. Exclusion of the open-air school slightly altered the findings. Larger longitudinal studies with improved exposure assessment are needed to clarify the effects of PM exposure and the influence of (open-air) school environments on children’s learning and development.

## 1. Introduction

The global population has been growing exponentially as well as anthropogenic sources of atmospheric pollutants. Urbanization and industrialization have led, and continue to lead, to the emission of high levels of air pollutants of which particulate matter (PM) is one of the key pollutants. PM is classified according to the aerodynamic diameter of its particles: PM_10_, PM_2.5_, PM_1_ and ultrafine particles (UFPs) are the main fractions. Combustion processes, particularly linked to traffic and industry emissions, are a major source. Nitrogen dioxide (NO_2_) is mainly derived from traffic and fuel combustion. As a common pollutant in cities, the highest NO_2_ levels are observed in urbanized and densely populated areas [1,2]. Hence, PM_2.5_ and/or PM_1_ and NO_2_ are frequently used as indicators of traffic-related pollution.

About 93% of the children worldwide are exposed to polluted air with levels exceeding international guidelines. This includes 630 million children under the age of 5 years and about 1.8 billion children under the age of 15 years [3,4]. In addition, children experience some of the greatest health impacts caused by air pollutants. As children are a uniquely vulnerable part of the population, their exposure to air pollution is of special concern as their immune system, neuropsychological abilities, and lungs are still developing [4]. In addition, children have a larger lung surface and breathe more air per mass of body weight, increasing pollutant intake compared to adults. They spend more (active) time outside during peak traffic hours and play closer to the ground, where PM is more concentrated, making them vulnerable to pollutants [3,5]. Importantly, this harmful exposure is not limited to PM or traffic-related pollution alone but can also stem from secondary pollutants such as ozone [6].

Strong evidence exists for the adverse health effects of exposure to PM. A large, and growing, body of scientific evidence has shown that short-term NO_2_ exposure can irritate the airways and aggravate existing respiratory conditions [3]. Previously suggested underlying mechanisms of pollutants on health include oxidative stress and pulmonary or systemic inflammatory responses, provoking endothelial dysfunction, which is an early predictor of cardiovascular disease [7,8]. The blood vessel endothelium, lining the inside of our blood vessels, is an interesting target for air pollution since the smallest particles (PM_2.5_, PM_1_, and UFPs) are able to enter the bloodstream. Exposure to traffic-related air pollution, and UFPs in particular, has been linked to a decrease in endothelial function (EF) in adults [9,10] and children [11,12,13]. However, evidence in children remains limited.

Cognitive functions, including executive function (such as working memory and attention), are essential for learning and achievement. These functions develop significantly during childhood, especially at primary school age [14]. There is a growing body of epidemiological research that found detrimental effects of air pollution on (children’s) cognition [5,14]. Furthermore, disrupted neurodevelopment due to air pollution exposure can lead to lower cognitive test outcomes [3,4]. Despite the amount of research, variation in study designs still leads to inconsistent results. Endothelial dysfunction could be suggested as a primary mediator when it comes to the impact of pollution on neurocognition.

Children spend a considerable amount of time in school, up to 8 h (a third) of their day. During school hours, traffic-related air pollution often exhibits peaks associated with morning and afternoon commuting and schools might be located near busy roads or other local pollution sources [15]. Therefore, standard (or national) pollution measurements, commonly used in epidemiology, may not provide an accurate estimation of children’s exposure [16,17]. There is an ongoing need for accurate exposure assessment of children, and the school environment is an important factor to consider in this case. Poor air quality conditions in school environments could impact children’s learning progression and cognitive performance, which may have an impact on their future quality of life with economic implications for society. Therefore, the goal of this study was to evaluate the endothelial function and neurocognitive abilities of healthy children in response to their PM exposure at the school level in a longitudinal study design.

## 2. Materials and Methods

### 2.1. Study Area and Population

**Figure 1:**
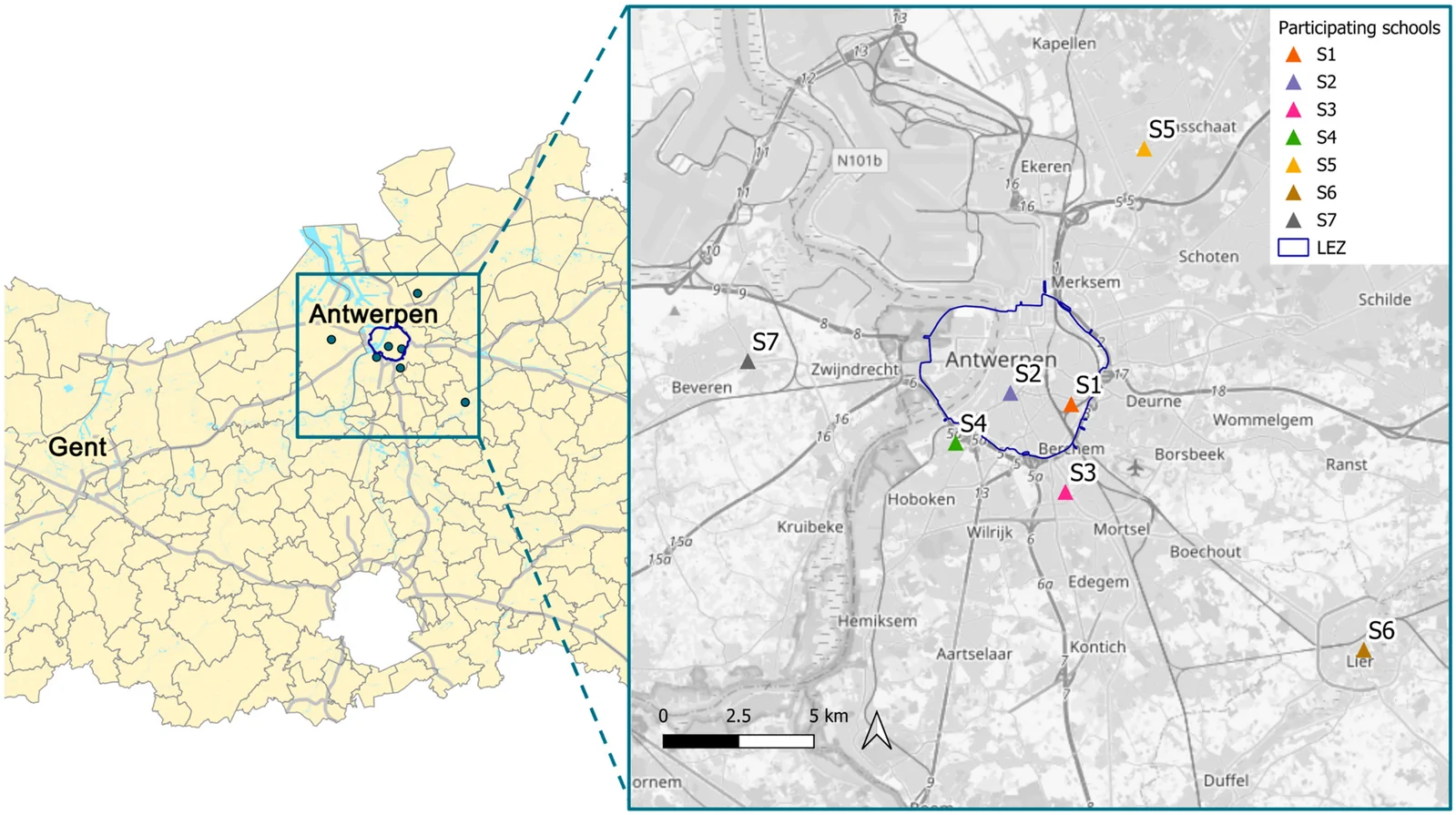
Participating schools. Study area with specific location of the participating schools (blue dots on the left side, S1–S7 on the right). LEZ = Low emission zone. Source background map (**left**): Grootschalig Referentie Bestand (GRB) Vlaanderen, Digitaal Vlaanderen (Agentschap Digitaal Vlaanderen), reproduced under the Flemish Government Open Data License.

This study was organized in and around the city of Antwerp, Belgium. Seven schools participated in the study: four of them were located within the Agglomeration of Antwerp near the city center and the ring of Antwerp (S1–S4, Figure 1) and three were located outside the agglomeration (S5–S7, Figure 1) with one of them in the zone of the Port of Antwerp (S7, Figure 1), an area which is characterized by substantial industrial and freight transport activity. Antwerp is a densely populated urban area with important traffic corridors, including the ring road and major road networks, which contribute to spatial variability in air pollutant concentrations. In 2017, a low-emission zone (LEZ) was implemented in the city center to reduce emissions from the most polluting vehicles. Two of the participating schools are situated within the LEZ (S1 and S2). One of the schools (S5) follows the open-air school principle, a school concept in which classrooms are designed to provide continuous exposure to outdoor air by keeping one wall of the classroom consistently open. Consequently, ventilation conditions and indoor–outdoor air exchange at this school differ from those of conventional school buildings, which may influence measured indoor pollutant concentrations.

Healthy children attending the schools, aged 10–12 years, were invited to participate between October 2022 and June 2023. Written informed consent was obtained from all children and their parents or legal guardians. This study was approved by the Ethics Committee of the University Hospital Antwerp (Project ID 3145—BUN B3002022000014).

### 2.2. Study Design

This is a longitudinal panel study with a repeated-measures design, in which each participant was followed over the course of one school year. Air pollution concentrations were measured continuously in the participating schools. Meanwhile, on-site study visits were planned throughout this period to measure health outcomes by a team of trained staff that visited each school three times (October 2022, February 2023, and May 2023). The study was designed to assess short-term exposure effects, with exposure windows of 4 h during the study visit (direct exposure), as well as 24 h (acute exposure) and 1 week (recent exposure) preceding the study visits for PM and a 4-week average exposure for NO_2_. Participants in school S2 were spread over two days for each visit. All measurements were non-invasive and occurred at the school site. Visits were made in the morning on a standard school day. Participants were in a fasted state until after the endothelial function measurements. A total of 138 children from the seven schools participated in this study. No formal a priori power calculation was performed as the study was exploratory in design. Further details on air pollution measurements are provided in Section 2.4.

### 2.3. Clinical Data Collection

At the beginning of all three visits, the height and weight of each participant were measured using a standing stadiometer and digital weighing scale. The body mass index (BMI) was calculated and further analyzed as BMI z-scores using the Flemish Growth Study as a reference population [18]. Blood pressure was measured three times using a validated oscillometric device with an adjusted cuff, and the average blood pressure of these three measurements was used. Pulse pressure, a measure of artery stiffness and flow pulsatility, was calculated as the difference between systolic and diastolic blood pressure [19].

#### 2.3.1. Questionnaires

A questionnaire was sent to the parents of the participating children to collect information on socioeconomic status, passive smoking, and living environments. The ISAAC (International Study of Asthma and Allergies in Childhood) questionnaire was used to identify possible asthma and allergic symptoms [20]. The questionnaire data were used solely to evaluate possible exclusion criteria. Due to a low response rate, these variables were not included in the statistical analyses.

#### 2.3.2. Endothelial Function

Due to the substantial time required for the assessment, peripheral microvascular endothelial function was measured in a subset of participants (*n* = 121). For these exploratory analyses, no separate a priori sample size calculation was performed.

Endothelial function was measured non-invasively using reactive hyperemia peripheral arterial tonometry (RH-PAT) with the Endo-PAT 2000 (Itamar Medical Ltd., Caesarea, Israel). This technique involves a plethysmographic evaluation of small arterial pulsatile changes on the fingertip. Children were in supine position on the examination table during the measurement. Pneumatic probes were placed at the distal phalanx of their index fingers on both hands. During the first 5 min, baseline measurements were taken at rest. This was followed by 5 min of forearm ischemia induced by inflating a blood pressure cuff on the non-dominant arm to 60 mmHg suprasystolic pressure. The reactive hyperemia response was then measured for another 5 min after deflating the cuff. Measurements were performed according to previously published recommendations [21]. The following parameters were extracted for data analysis: Reactive Hyperemia Index (RHI), the time-to-peak response (in seconds) and the maximal dilatation. Quality control was performed manually using the Endo-PAT 2000 software (version 3.6.2). Measurements of poor quality were excluded.

#### 2.3.3. Neurocognitive Functions

Neurocognitive functions, focusing on attention, were tested via computer-assisted screening tests using the E-Prime software (Version 3.0.; Psychology Software Tools, Pittsburgh, PA, USA). The Continuous Performance Test (CPT) was used to test sustained attention and impulse control [22]: letters were presented every 1000 ms on a screen, and the subjects were asked to push the space bar on a keyboard when ‘X’ appeared. This lasted 7 min. Furthermore, the Stroop test was used for selective attention and inhibitory control [23]. The words yellow, red, blue, and green were shown in matching colors (congruent condition) and non-matching colors (incongruent condition). First, subjects responded by pushing the button corresponding to the color in which the words were displayed. Second, the subjects responded by pushing the button matching the word displayed on the screen. The mean response time (msec) and accuracy (%) were calculated for each test per participant for further data analysis.

### 2.4. Air Pollution Exposure

Air pollution concentrations were continuously measured. At each school, real-time monitoring equipment was installed indoors (MX-KIT020 mini XT basic, Ethera Labs, Grenoble, France) and outdoors (NE-OAM065 NEMo^®^ outdoor, Ethera Labs, Grenoble France). These devices estimate particulate matter concentrations using a laser light-scattering optical particle counter (including temperature and humidity adjustments for outdoor measurements), which estimates PM mass concentrations from the size distribution and number concentration of airborne particles using manufacturer-specific algorithms. Indoor concentrations (µg/m^3^) of CO_2_, PM_2.5_, temperature (°C), relative humidity (RH%), and air pressure were measured inside the classroom. Indoor monitors were placed on a desk or classroom cabinet. Outdoor pollutant concentrations (µg/m^3^), including PM_10_, PM_2.5_, and PM_1_, were measured in the playground. Outdoor monitors were installed at a height of 1.5–2 m. Data were logged every 10 min and were accessible through the NEMo^®^ cloud. All devices were factory-calibrated by the manufacturer prior to deployment. No additional gravimetric calibration was performed on site. Air pollution data were cleaned prior to analysis. Values greater than 10 and twice the average of the preceding and subsequent values were replaced with the average of those neighboring values. Data collected outside school hours, on weekends or during school and public holidays, were excluded. For each school, the following exposure estimates were calculated per pollutant: median and the 90th and 95th percentile (P90 and P95) for 4 h during the study visits and for the 24-h and 1-week periods preceding the study visits. The time windows were defined as direct (4 h), acute (24 h before), and recent (1 w before) exposure. Only air pollution data linked to visit two and three were available.

Additionally, passive monitoring, using acrylic Palmes diffusion tubes, was used to collect NO_2_ data. Two tubes were installed per sampling point at different locations on the school grounds. The mean of the two samples per location was calculated to obtain the concentrations. The tubes were replaced monthly, after approximately four weeks of air sampling. Afterwards, they were sent to an ISO/IEC 17025-accredited laboratory (Buro Blauw, Wageningen, The Netherlands) for analysis (absorption spectrophotometry) [24]. An accredited method conforming to NEN-EN 16339 was used to determine the 4-week average NO_2_ concentrations (µg/m^3^). The NO_2_ concentrations corresponding to the month of each school visit were used to estimate exposure. Subsequently, 4-week NO_2_ concentrations were related to the real-time PM and CO_2_ measurements by aggregating the latter over the corresponding NO_2_ sampling periods, which is described further in Section 2.5.

### 2.5. Statistical Analysis

Categorical data are summarized as numbers and percentages. For continuous data, normality was assessed through the visual inspection of QQ-plots, combined with the Shapiro–Wilk test. Data are summarized as the mean with the standard deviation (SD) in the case of normality and as the median with the lower and upper quartile (Q1–Q3) otherwise. Evolution of blood pressure, endothelial function, and neurocognition over time was studied using linear mixed-effects models with fixed effects for the visit (visits 1–3), baseline age, sex, and BMI z-score and a random intercept for children nested within schools. If the effect of visit was significant, model-based post hoc pairwise comparisons between the three visits were performed.

To investigate the association between monthly NO_2_ measurements and real-time air pollution data (e.g., outdoor PM_10_, PM_2.5_, and PM_1_ and indoor PM_2.5_), the real-time pollutant concentrations were aggregated over the corresponding NO_2_ assessment periods. For each school and assessment period, the median of the real-time measurements was computed. Separate linear mixed-effects models were then fitted for each pollutant, with the NO_2_ concentration as the outcome, the median pollutant concentration as a fixed effect, month as a fixed factor, and a random intercept per school to account for repeated measurements within schools.

To assess the impact of air pollution exposure on blood pressure, endothelial function, and neurocognition, a linear mixed-effects model was fitted with a summary of one of the air pollution variables as a fixed effect (outdoor PM_10_, PM_2.5_, and PM_1_ and indoor CO_2_ and PM_2.5_). As a summary, we considered the median and P90 and P95 concentrations of the 4 h during the visit and those preceding each visit by 24 h and 1 week. Additionally, visit (2 or 3), baseline age, sex, and BMI-z were included in the model as fixed effects, and a random intercept for children nested in schools was added to account for within-subject variability. Similarly, for each of the outcome measures, a linear mixed-effects model was fitted with the 4-week average NO_2_ result in the month of the visit, visit (1–3), age, sex, and BMI-z as a fixed effect and random intercept for children within schools. As temperature can influence endothelial function measurements, all analyses for endothelial function were repeated with room temperature added as a fixed effect to the models described above. Model assumptions were evaluated via visual inspection of residual plots. When necessary, logarithmic transformation of variables was applied to meet model assumptions. Effect estimates are presented as standardized population-level (fixed effect) regression coefficients (β), with standard error (se), 95% confidence intervals (CIs), and corresponding *p*-values. All statistical analyses were performed in R version 4.5.1. A two-sided *p*-value < 0.05 was considered statistically significant.

## 3. Results

### 3.1. Study Population, Neurocognition, and Endothelial Function

A total of 154 healthy children were initially included, of whom 138 ultimately participated in the study. On the first study visit, 21 children were absent from school. Of these children, 16 were excluded, while 5 were enrolled during one of the next visits. The number of participants per school and their main demographic and anthropometric characteristics can be found in Table 1. The proportion of boys at baseline was 55.8% and the mean age (SD) at baseline was 10.3 (0.5) years. Except for one participant with asthma (based on the questionnaires), no comorbidities were reported.

#### Descriptive Statistics

Over three school visits, a total of 264 endothelial function measurements were obtained from 121 of the 138 enrolled participants. Within the total enrolled sample, 51 participants (37%) had measurements taken on all three visits, and 41 (30%) had measurements in two out of three visits. Additionally, 29 participants (21%) only completed a single measurement, while in 17 participants (12%), endothelial function was not assessed. No significant difference in RHI or time-to-peak response was observed over time (Table 2). The maximal dilatation was significantly lower on the third visit, compared to the second, whereas blood pressure was the highest on the second visit compared with visits 1 and 3 (Table 2).

For neurocognitive testing, a total of 332 measurements were obtained from 129 different participants. Overall, 84 participants (61%) completed the tests on all three visits, while 34 participants (25%) participated during two out of three visits. Additionally, 12 participants (8%) completed only a single visit, and 8 participants (6%) did not undergo neurocognition testing. There was a significant difference in response time and accuracy between visits (Table 2). A decrease in response time was observed on the third visit compared with both the first and second visit, for the Stroop test and CPT. Accuracy improved for the Stroop task and CPT, with significant differences observed on visits two and three compared with on visit one (Table 2).

### 3.2. Air Pollution

#### 3.2.1. Real-Time PM Concentrations

**Figure 2:**
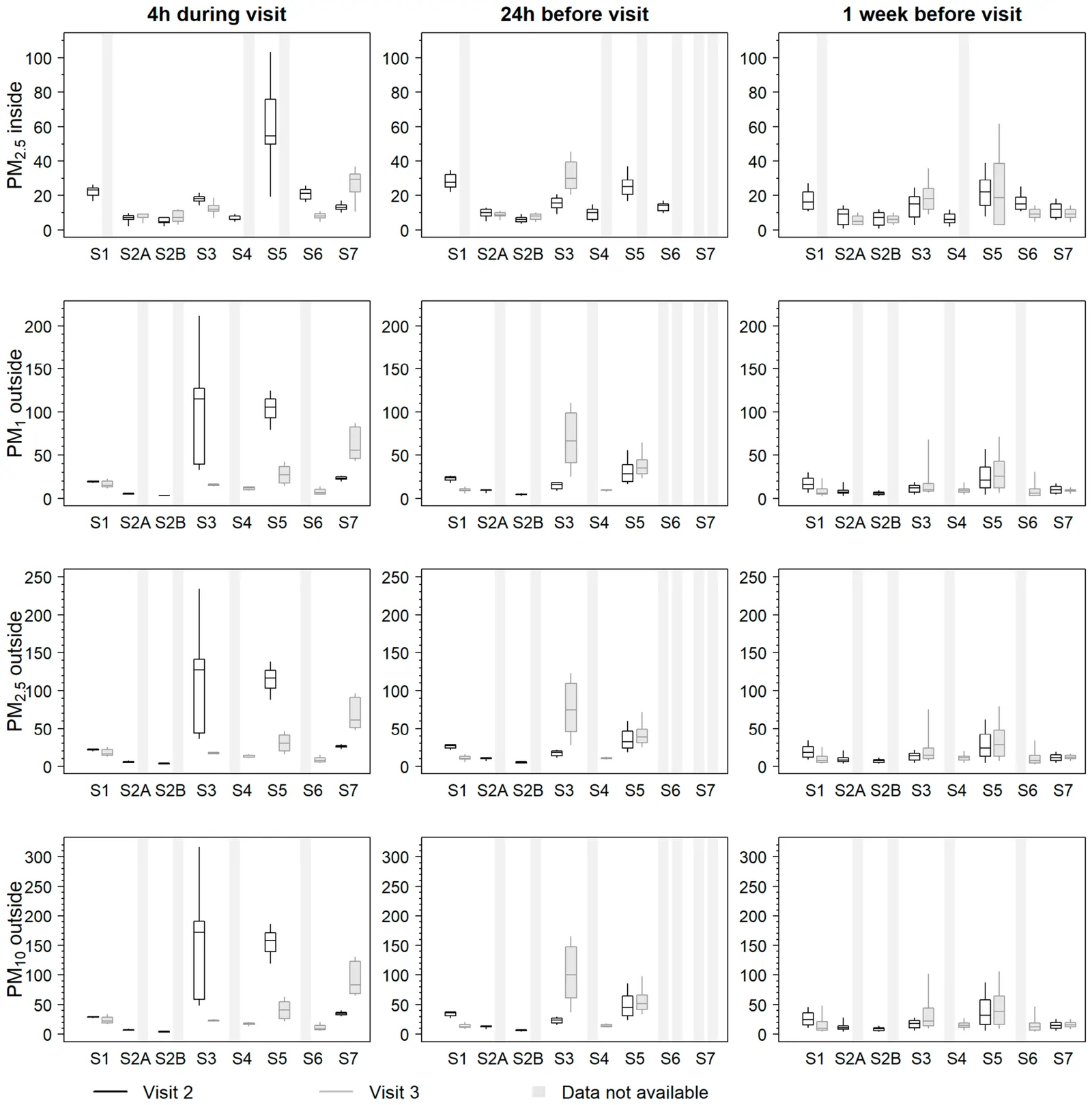
Indoor PM_2.5_ and outdoor PM_1_, PM_2.5_, and PM_10_ concentrations preceding visits two and three. Percentile boxplots of PM concentrations are shown for each exposure window per school, linked to the second and third visits (black/white boxplots for visit 2 and grey boxplots for visit 3). Whiskers represent P10–P90 range. Participants in school S2 were spread over two days for each visit (S2A and S2B). PM concentrations are expressed in µg/m^3^.

Real-time PM_2.5_ was measured indoors and real-time PM_1_, PM_2.5_, and PM_10_ were measured outdoors. These air pollution variables are shown in Figure 2 per school, for the 4-h exposure window during and the 24-h and 1-week windows preceding the second and third visits.

The open-air school (S5) showed the greatest variability and the highest peak values in indoor PM_2.5_, particularly for the 4-h window during the second visit. Outdoor PM_2.5_ concentrations showed a similar pattern in the open-air school, mainly for the second visit, although school S3 also exhibited a large variability in outdoor PM_2.5_. The highest peak values for outdoor PM_2.5_ were measured in schools S3 and S5.

#### 3.2.2. Monthly NO_2_

**Figure 3:**
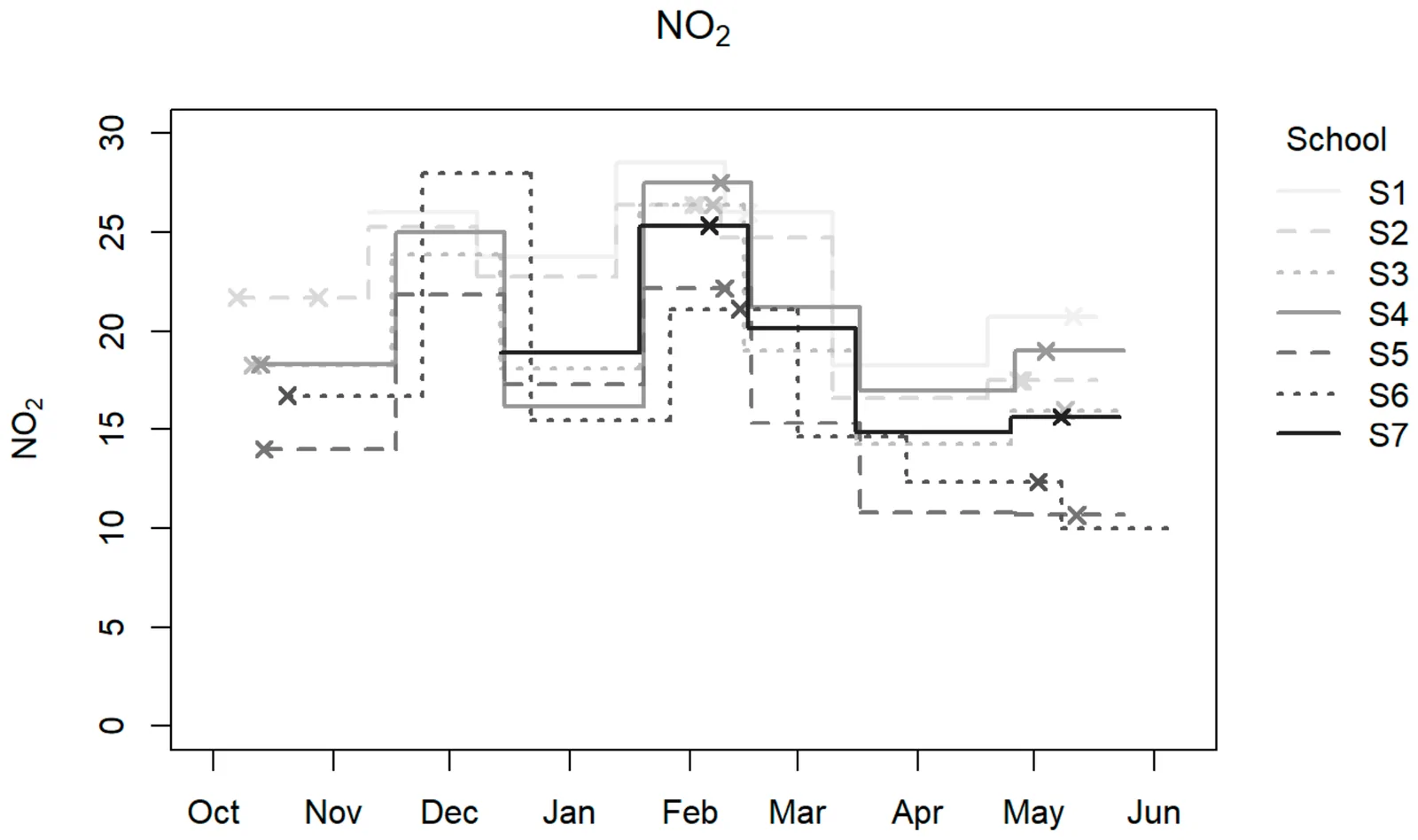
Monthly average NO_2_ concentrations across the different schools. School S1–S7 are depicted, of which S1–S4 are city-centered schools and S5 is the open-air school. The exact location of the different schools can be found in Figure 1. Each study visit is indicated as X on the graphs. NO_2_ = average NO_2_ concentrations (µg/m^3^).

NO_2_ measurements were started at different time points across the participating schools (Figure 3). NO_2_ concentrations linked to the first visit were therefore unavailable for schools S1 and S7. Both the month of sampling (F(6, 33) = 31.79, *p* < 0.001) and school (F(6, 33) = 13.31, *p* < 0.001) had a significant effect on NO_2_ concentrations.

NO_2_ levels were the highest during the winter period (November–February; *p* < 0.001) and the lowest during the spring period (March–May; *p* < 0.05), compared to October as the reference month. Consequently, NO_2_ concentrations were higher during the second visit (February 2023) compared to the first and third visits. Across schools, the highest NO_2_ concentrations were observed at one of the city-center schools (S1, Figure 1). All other schools exhibited significantly lower NO_2_ concentrations compared to school S1 (all *p* < 0.01), except for school S2. NO_2_ concentrations were significantly correlated with real-time indoor PM_2.5_ (*p* = 0.037) but not with outdoor pollutant concentrations (PM_1_, PM_2.5_, and PM_10_; *p* = 0.53, *p* = 0.50, and *p* = 0.50).

### 3.3. Association Between Air Pollution and Health Outcomes

#### 3.3.1. Blood Pressure and Endothelial Function

**Figure 4:**
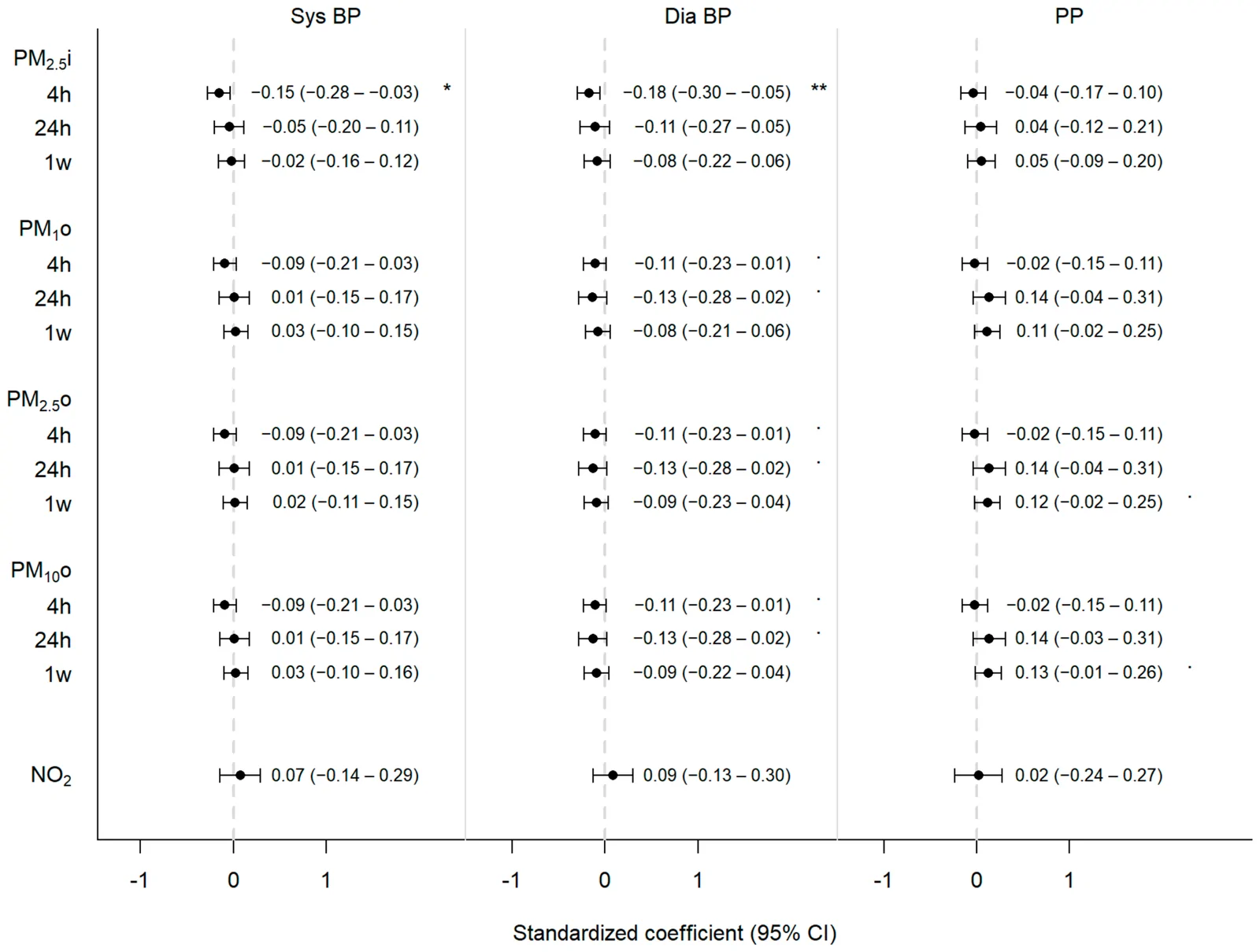
Associations between blood pressure parameters and air pollutant concentrations. Linear mixed models were fitted separately for each air pollutant variable and adjusted for visit, age, sex, and BMI z-score. Sys BP = Systolic blood pressure; Dia BP = diastolic blood pressure; PP = pulse pressure. PM_2.5_ i = indoor PM_2.5_; PM_1_ o, PM_2.5_ o, PM_10_ o = outdoor PM. Statistical significance is indicated as: *** = *p* < 0.001, ** = *p* < 0.01, * = *p* < 0.05, and · = *p* < 0.1.

Significant associations were observed between blood pressure measures and real-time indoor PM_2.5_ concentrations (Figure 4). Direct exposure (4 h) was inversely associated with both diastolic and systolic blood pressure independent of sex, age and BMI-z. In addition, systolic blood pressure was positively associated with the highest levels (P95) of recent outdoor PM_1_ (*p* = 0.037) and PM_2.5_ (*p* = 0.044). A similar pattern was observed for pulse pressure, which was positively associated with the highest concentrations (P90 and P95) of recent outdoor PM_1_ (*p* = 0.007; *p* = 0.005), PM_2.5_ (*p* = 0.007; *p* = 0.005), and PM_10_ (*p* = 0.008; *p* = 0.005). Detailed results of the linear mixed models, including standardized coefficients, standard errors, and corresponding *p*-values, are provided in Supplementary Table S1.

**Figure 5:**
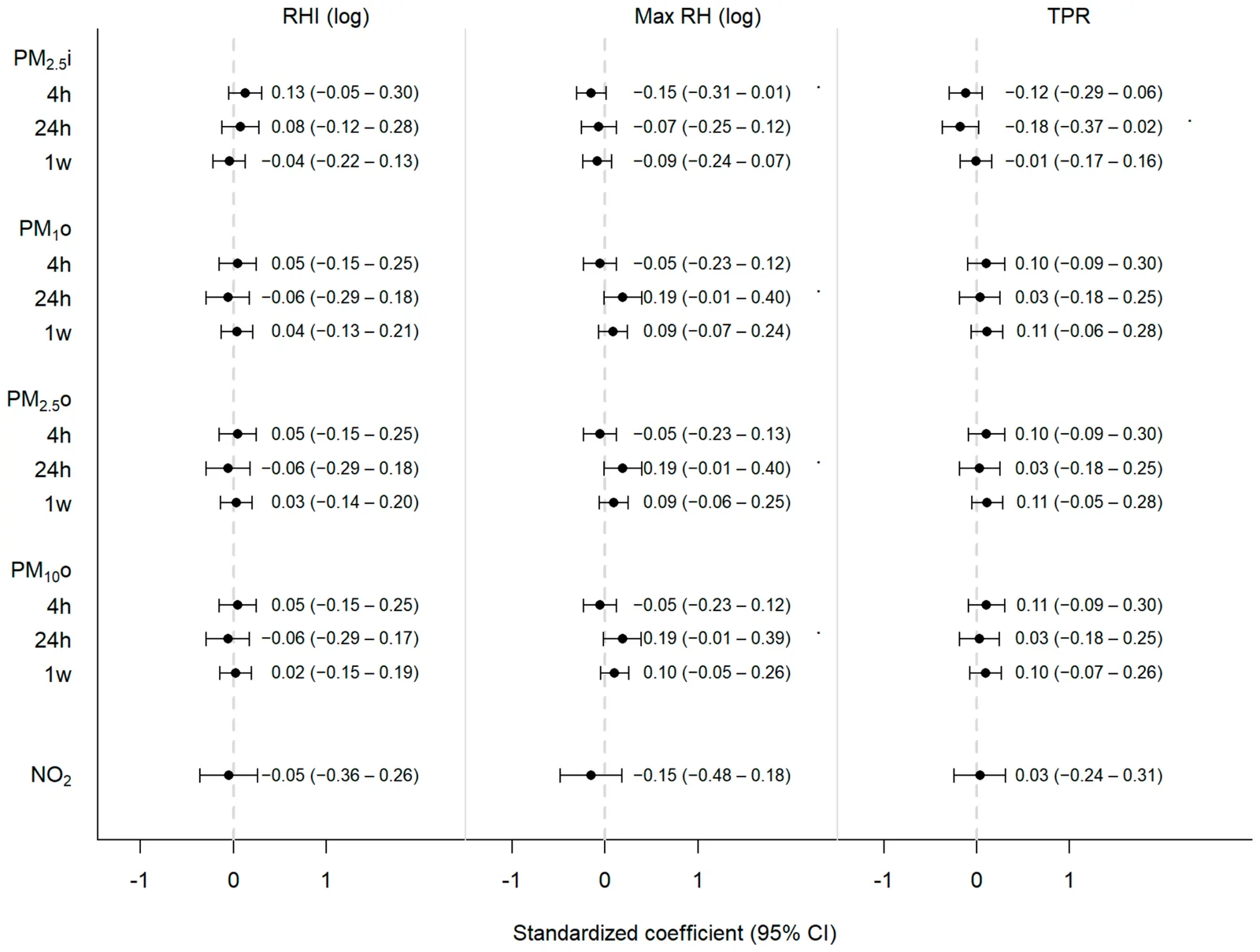
Associations between endothelial function parameters and air pollutant concentrations. Linear mixed models were fitted separately for each air pollutant variable and adjusted for visit, age, sex, BMI-z, and temperature. RHI = Reactive Hyperemic Index; Max RH = maximal dilatation; tpr = time-to-peak response. PM_2.5_ i = indoor PM_2.5_; PM_1_ o, PM_2.5_ o, PM_10_ o = outdoor PM. Statistical significance is indicated as: *** = *p* < 0.001, ** = *p* < 0.01, * = *p* < 0.05, and · = *p* < 0.1.

Endothelial function was associated with direct indoor PM_2.5_ levels after adjusting for sex, age, and BMI-z, with the maximal dilatation decreasing as levels of indoor PM_2.5_ increased (Figure 5). However, the association was only significant for the highest indoor PM_2.5_ values (P90 and P95, *p* = 0.047 and *p* = 0.045). In contrast, no associations were observed between endothelial outcomes and outdoor PM. As temperature can influence endothelial function measurements, the analyses were repeated with a correction for room temperature; however this did not alter our findings. No associations were found between any cardiovascular parameters and 4-week average NO_2_ concentrations. Detailed results of the linear mixed models for endothelial function, including standardized coefficients, standard errors, and corresponding *p*-values, are provided in Supplementary Table S2.

#### 3.3.2. Neurocognition

**Figure 6:**
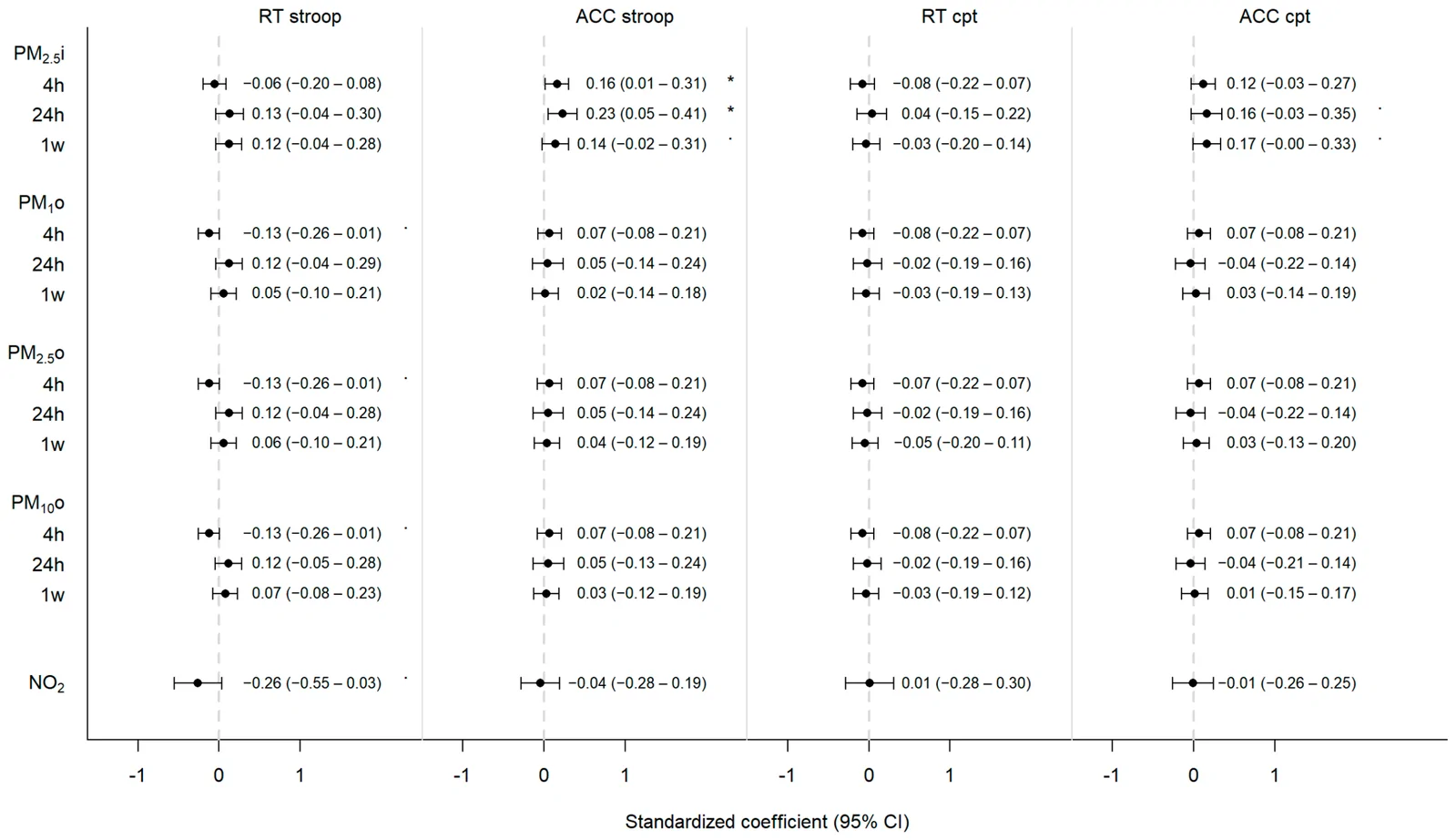
Associations between neurocognitive function parameters and air pollutant concentrations. Linear mixed models were fitted separately for each air pollutant variable and adjusted for visit, age, sex, and BMI-z. RT = Response time; ACC = accuracy. Stroop task is for selective attention measurement; CPT = continuous performance test for sustained attention measurement. PM_2.5_ i = indoor PM_2.5_; PM_1_ o, PM_2.5_ o, PM_10_ o = outdoor PM. Statistical significance is indicated as: *** = *p* < 0.001, ** = *p* < 0.01, * = *p* < 0.05, and · = *p* < 0.1.

No clear associations were observed between PM concentrations and the response time for either the Stroop task or CPT, after adjusting for sex, age, and BMI-z (Figure 6). However, the 4- and 24-h windows of indoor PM_2.5_ exposure were associated with increased accuracy in selective attention (Stroop test). In addition, the accuracy of sustained attention (CPT) increased with recent indoor PM_2.5_ (one week before the visit), although only significant for the highest values (P95, *p* = 0.037). No associations were observed with outdoor PM or NO_2_ concentrations. Detailed results of the linear mixed models, including standardized coefficients, standard errors, and corresponding *p*-values, are provided in Supplementary Table S3.

### 3.4. Open-Air School Environment

**Figure 7:**
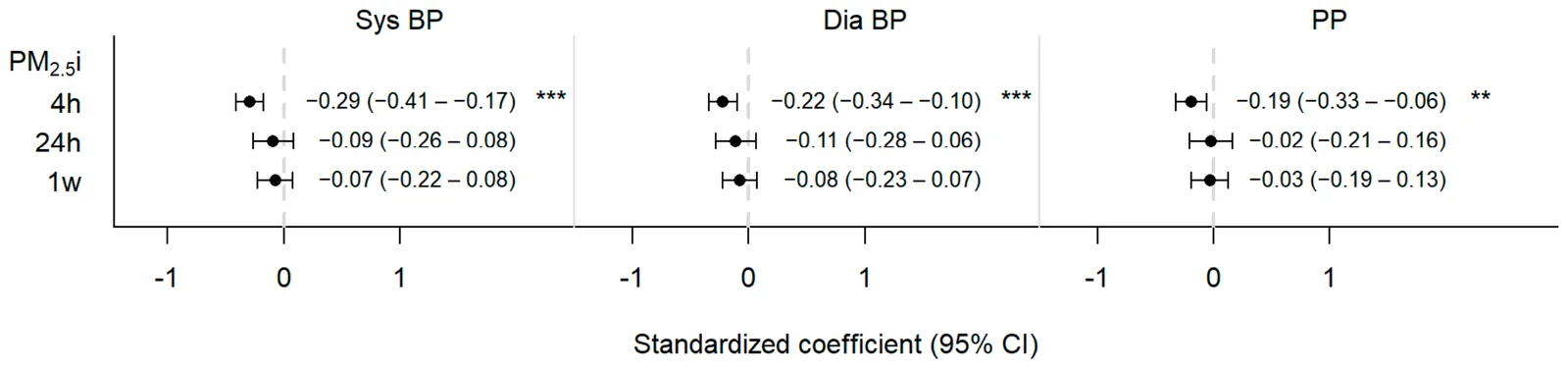
Associations between blood pressure parameters and indoor PM_2.5_ without open-air school. Analysis was repeated without the open-air school. Linear mixed models were fitted for indoor PM_2.5_ variables and were adjusted for visit, age, sex, and BMI-z. Sys BP = Systolic blood pressure; Dia BP = diastolic blood pressure; PP = pulse pressure. Statistical significance is indicated as: *** = *p* < 0.001, ** = *p* < 0.01, * = *p* < 0.05, and · = *p* < 0.1.

**Figure 8:**
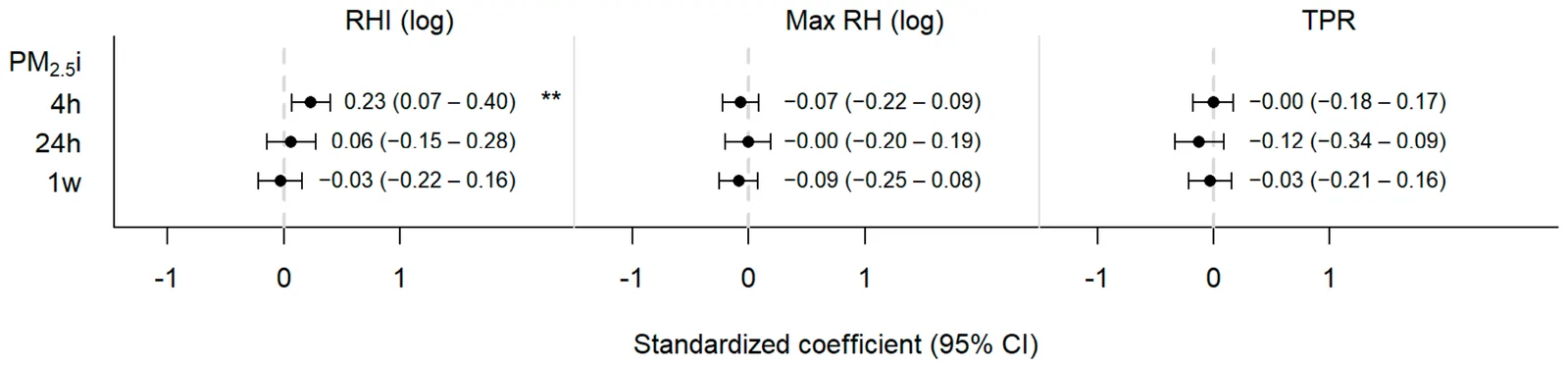
Associations between endothelial function parameters and indoor PM_2.5_ without open-air school. Analysis was repeated without the open-air school. Linear mixed models were fitted for indoor PM_2.5_ variables and were adjusted for visit, age, sex, BMI-z, and temperature. RHI = Reactive Hyperemic Index; Max RH = maximal dilatation; tpr = time-to-peak response. Statistical significance is indicated as: *** = *p* < 0.001, ** = *p* < 0.01, * = *p* < 0.05, and · = *p* < 0.1.

Considering the open-air principle in school S5, this school could be considered an outlier for indoor air pollution measurements. We therefore repeated all indoor analyses without school S5. This revealed an inverse association between direct PM_2.5_ exposure and pulse pressure, while the previous negative associations found with systolic and diastolic blood pressure persisted (Figure 7). In addition, RHI was positively associated with direct indoor PM_2.5_ concentrations, whereas the previous association found with maximal dilatation did not persist (Figure 8).

**Figure 9:**
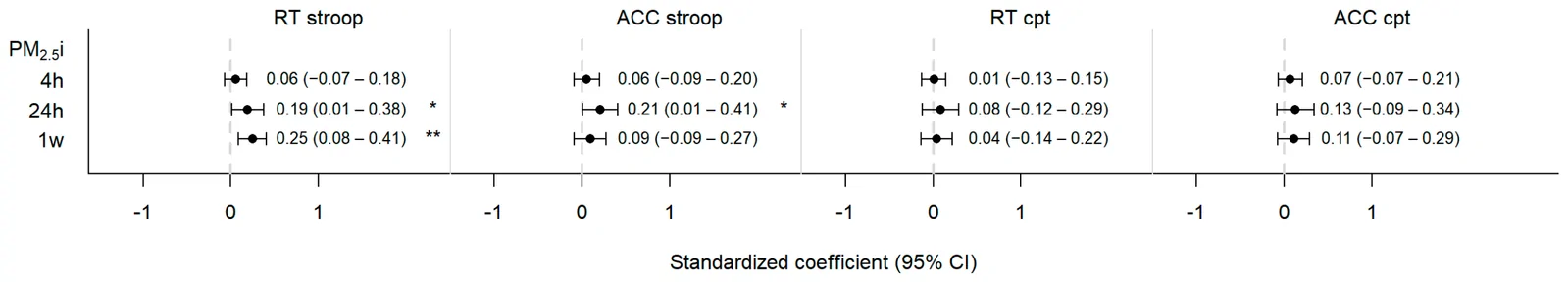
Associations between neurocognitive function parameters and indoor PM_2.5_ without open-air school. Analysis was repeated without the open-air school. Linear mixed models were fitted for indoor PM_2.5_ variables and were adjusted for visit, age, sex, and BMI-z. RT = Response time; ACC = accuracy. Stroop task is for selective attention measurement; CPT = continuous performance test for sustained attention measurement. Statistical significance is indicated as: *** = *p* < 0.001, ** = *p* < 0.01, * = *p* < 0.05, and · = *p* < 0.1.

For the neurocognitive outcomes, slower response times were found for selective attention with increasing direct (24 h) and recent (1 w) PM_2.5_ exposure. The previous associations with selective attention accuracy only persisted with acute (24 h) exposure, while the association with CPT accuracy did not persist (Figure 9).

## 4. Discussion

In this longitudinal study, we investigated the effect of air pollution in the school environment on the cardiovascular system and neurocognitive abilities of healthy children. In general, indoor PM concentrations showed minimal negative associations with endothelial outcomes and blood pressure, while positive associations were observed with neurocognitive outcomes. No associations were observed for outdoor PM, except for a positive link with blood pressure.

Air pollution was measured at the school level, inside the classroom, and outside on the playground. The highest peak levels of indoor PM_2.5_ were observed in the open-air school (S5), which is expected given the design of the school, where one wall of the classroom is consistently kept open. High outdoor PM_2.5_ levels were also measured at this school. We did not observe this pattern for NO_2_ levels. However, the degree of urbanization, traffic density, and distance to a nearby highway are known to significantly influence outdoor NO_2_ levels [2]. This may partially explain our findings as the open-air school was situated in a more suburban area. Indeed, the highest NO_2_ levels were observed in a city-centered school (S1) in a highly urbanized area with closely spaced buildings where dispersion of pollutants may be reduced due to a moderate street-canyon morphology. The presence of a nearby public transportation bus depot may contribute to increased traffic volumes in the vicinity of the school. A seasonal pattern was also observed at the school-site NO_2_ measurements, with higher concentrations in colder months and lower levels in spring/summer, which is consistent with expected variability in traffic- and residential heating-related emissions and seasonal changes in atmospheric mixing conditions.

There is a growing body of evidence suggesting the role of air pollutants in the early disruption of blood pressure regulation, as summarized by Manuel et al. [25]. Even though they identified air pollution as a significant determinant of childhood hypertension, we could not entirely confirm this result as we found negative associations between indoor PM_2.5_ levels and blood pressure, and no link with 4-week average NO_2_ levels. However, a positive association between pulse pressure and outdoor PM was identified, which could suggest increased arterial stiffness or impaired vascular function, especially combined with an increased systolic pressure in our population. Air pollution, including PM_2.5_ and NO_2_, has also been linked to endothelial dysfunction, oxidative stress, and systemic inflammation, which can all impair vascular homeostasis and contribute to elevated blood pressure [7,8,25]. Oxidative stress is induced through increased production of reactive oxygen species (ROS), leading to reduced endothelial nitric oxide (NO) bioavailability, reduced vasodilatory capacity, increased endothelin-1 (ET-1) levels, and increased arterial stiffness [7]. The capacity of PM to induce oxidative stress is closely related to its oxidative potential (OP), which is a measure of the ability of particles to produce ROS or deplete antioxidants in biological fluids, offering a more biologically relevant measure of PM toxicity than particle mass alone [26]. OP reflects the effects that the interaction among the PM chemical composition, physical properties, and possible toxicological effects may induce on human health [27]. Combustion- and traffic-related particles exhibit higher OP due to enrichment with transition metals and redox-active organic compounds, which may contribute to their adverse effects [28]. Several in vitro studies have found that PM OP is associated with intracellular ROS generation, oxidative damage, and antioxidant responses, although findings have been inconsistent across different health outcomes [29]. Furthermore, epidemiological evidence indicates that short-term exposure to PM with higher OP is more strongly associated with adverse cardiorespiratory outcomes than PM mass concentration alone, supporting its relevance as a health-based exposure metric [30,31]. In parallel, PM exposure has been associated with the upregulation of pro-inflammatory cytokines, such as high-sensitive C-reactive protein (hs-CRP), which may further exacerbate vascular inflammation and dysfunction [32]. Supporting these mechanisms, Kelishadi et al. reported that PM_10_ exposure was associated with decreased serum NO levels and an increased hs-CRP in a healthy pediatric population [33]. Studies by Calderon-Garciduenas and colleagues reported a link between PM_2.5_ exposure and increased ET-1 in children [11,34]. These mechanisms would be expected to result in an impaired endothelial function with higher pollution exposure. In our study, endothelial function was indeed adversely related to direct indoor PM_2.5_ exposure, with maximal dilatation (which reflects the greatest extent of vasodilation observed during the reactive hyperemia phase) decreasing as indoor PM_2.5_ levels increased, indicating an impaired vasodilatory response with higher indoor pollution levels. Overall, the absence of longitudinal changes in RHI and tpr suggests that microvascular endothelial function remained largely stable across visits. The lower maximal dilatation observed on visit 3, in the absence of parallel changes in RHI, may reflect differences in baseline peripheral vascular tone rather than a true alteration in reactive hyperemic capacity. Given that visit 3 took place in May, higher ambient and room temperatures may have contributed to peripheral vasodilation at baseline. In contrast, the higher blood pressure observed on visit 2, conducted in February, is consistent with the described seasonal variation in blood pressure in children, with higher values during colder periods likely related to sympathetic activation and peripheral vasoconstriction [35,36].

In another study, by Hashemi et al., air pollution and passive smoking were linked with an impaired endothelial function, measured via flow-mediated dilatation (FMD) in healthy children [12]. In contrast to these findings and our results for maximal dilatation, we observed a positive association between direct indoor PM_2.5_ and RHI when the open-air school was excluded. This contradictory pattern may reflect that RHI and maximal dilatation capture partly different aspects of endothelial function, respond differently to short-term exposure peaks, and are also likely influenced by unmeasured confounding factors (for example, different underlying participant characteristics). In our study, microvascular endothelial function was studied using RH-PAT, which enabled us to investigate the dynamic response and elasticity of the small vessels. In contrast, FMD studies the macrovascular endothelial function [37,38]. Furthermore, while no association was found between FMD and age, RHI has been shown to be age-dependent [37]. This is important to consider when interpreting results in children. In adults, an RHI score < 1.67 is considered abnormal [21], while other studies suggest a cutoff value of 1.35 to identify endothelial dysfunction [37]. However, currently no reference values for children are available. In our pediatric population, the mean RHI was 1.27 ± 0.40, which is much lower than the proposed cut-off value in adults. This is in line with previous studies where RHI values were lower in the pediatric population compared to adults [37,38]. Indeed, RHI has been shown to increase with age and pubertal development in children and adolescents [37,38,39]. In line with the literature, we also observed substantial variability in measured RHI values [37,38,39]. To the best of our knowledge, no other studies have used the EndoPAT technique to measure endothelial function directly, in response to air pollution exposure in a healthy pediatric population.

Endothelial dysfunction could be suggested as an underlying mechanism for the effect of air pollution on the human brain. In agreement with this hypothesis, animal models have shown the possible neurotoxic effects of air pollution [40,41]. Furthermore, a study found that exposure to air pollution could lead to the breakdown of the blood–brain barrier in children [42]. The smallest particles can pass through the alveoli after inhalation and end up in the bloodstream, after which they can infiltrate the brain [43]. Likewise, particles can directly translocate to the olfactory bulb and migrate to the olfactory cortex, causing tissue damage and local inflammation after inhalation through the nose [5,43]. Inflammatory responses were also observed in the brain regions related to executive function after exposure to traffic-related air pollution in children and even structural changes were found in children’s brains [14,44,45].

A slower response time and increased error rates in incongruent trials, known as the Stroop effect, reflects interference from automatic word-reading processes. These factors are commonly used to measure selective attention and cognitive control [23]. Similarly, delayed responses and increased omission errors on the CPT indicate reduced sustained attention, while a higher rate of commission errors suggests increased impulsivity [22]. In this study, we evaluated the effects of air pollution on both selective (Stroop task) and sustained (CPT) attention. Overall, we found no associations between in- or outdoor PM and response times for either attention domain. Accuracy was found to increase with direct (4 h) and acute (24 h) indoor PM_2.5_ exposure for selective attention, while only the 95th percentile of recent indoor exposure (1 week) was associated with sustained attention. After excluding the open-air school, the associations between indoor PM_2.5_ and accuracy remained only for selective attention, while other associations with accuracy disappeared. In addition, this exclusion also revealed a positive association between acute (24 h) and recent (1 week) indoor PM_2.5_ levels and reaction time for selective attention. Our overall findings are in agreement with the results of Compa et al., where short-term PM_10_ exposure was associated with fewer omission errors in the CPT, without effects on the response time or selective attention outcomes [46]. However, after excluding the open-air school, our results align with those of Saenen et al., which reported that higher indoor PM_2.5_ and PM_10_ classroom levels were associated with longer response times in the selective attention domain, but not with sustained attention [47]. More recently, short-term fluctuations in air pollution have also been linked with acute changes in attention and cognitive performance [48].

Mixed findings on the impact of NO_2_ on cognitive outcomes have been reported in the literature. Previous studies have found that children exposed to higher short-term NO_2_ levels showed longer response times in selective attention tasks, combined with higher error rates [49,50]. In contrast, Compa et al. reported that short-term NO_2_ exposure was associated with decreased CPT response time. However, a trend towards more commission errors suggested more impulsive performance [46]. We could not confirm these results, as we found no associations between 4-week average NO_2_ and any of the measured cognitive outcomes.

Despite important strengths, this project has several limitations, which are mostly due to the restrictions of fieldwork in school environments and technical issues. To start, EndoPAT measurements are sensitive to several external factors [21]. Due to the field set-up, room temperature was not always within the recommended range (20–24 °C) and was therefore included in our statistical analyses to correct for this possible confounding effect. Additionally, EndoPAT quality was assessed by manual review, which may introduce assessor-dependent subjectivity. To reduce variability, all processing and scoring were performed by a single investigator. As a result, fewer (qualitative) endothelial measurements were available for analysis. Second, NCF assessments should ideally be conducted in a separate room, which was not always possible, leading to possible distractions during testing. Headphones were provided to limit these distractions as much as possible.

Next, due to technical issues with real-time air quality measurements, the dataset of PM concentrations was incomplete; so, only health outcomes from the second and third visits could be linked to real-time pollutant concentrations. Indoor PM was measured within a single classroom despite participation from multiple classrooms. Furthermore, NO_2_ was measured using passive diffusion tubes, providing integrated concentrations over a four-week period, rather than continuous measurements. This likely attenuated short-term (e.g., day-to-day) variability and masked peak concentrations. In addition, the 4-week average concentrations corresponding to the month of each school visit were used. However, the timing of the visits varied within the 4-week sampling period with some school visits falling at the beginning of this period and some at the end.

Additionally, due to low parental response rates for the questionnaires, it was not possible to include information on factors such as socioeconomic status, passive smoking, (active) time spent outdoors, or home environment in the analysis. Nevertheless, previous research found no effect of maternal education, parental occupation, passive smoking, or extracurricular sport activities on the associations between attention and PM concentrations [47], and PM_10_ was shown to affect endothelial function regardless of passive smoking [12,33]. In contrast, more time spent outdoors was linked to a slower selective attention response time [46]. Socioeconomic and environmental inequalities may influence certain pollution-related health effects. For example, Compa et al. found that children of parents with a higher education showed faster selective attention responses, but slower sustained attention responses with more correct rejections, illustrating how social factors can modify pollution-cognition associations [46].

It should also be noted that the inclusion of the open-air school might have influenced our results. Indeed, when excluding this school from the analysis between neurocognition and indoor PM levels, a positive association with response times was observed. However, neurocognitive performance may also be confounded by school-level characteristics such as socioeconomic context and site greenness, as the open-air school showed the highest level of greenery at its school site. Contact with green spaces, and their availability, has been shown to improve well-being, and mental and physical health [4,51]. Our study was conducted in the largely urbanized Antwerp region, with relatively homogeneous air pollution levels and no clear boundary between urban and rural areas, resulting in limited exposure contrasts between the schools, which may have limited effect detection. The modest sample size relative to the number of exposure–outcome associations examined, high variability within the health outcomes, and incomplete repeated measurements further reduced the analytical power of this study. As this study was designed as an exploratory study, no formal a priori power calculation was performed. The results should be interpreted with caution, as a large number of hypothesis tests were performed and no formal correction for multiple testing was applied. Consequently, the risk of type I error may be increased. However, given the exploratory nature of this study, we chose not to apply multiple testing corrections, as such procedures substantially increase the risk of type II error and may obscure potentially meaningful signals. Therefore, the findings should be considered hypothesis-generating and warrant confirmation in future studies.

Future studies should increase sample size and include more schools to detect more subtle effects. Furthermore, the potential (beneficial) effect of open-air school environments on the development of children should be explored further. A more detailed understanding of the air quality in and around school environments may contribute to improved strategies for reducing air pollution exposure in educational settings [15]. In the context of open-air schools, it is essential to consider the surrounding environmental conditions, including air pollution levels, level of greenery, and nearby pollution sources. Major roadways, industrial zones, and dense urbanization can significantly influence local air quality [2]. A key question is whether the implementation of open-air school environments remains beneficial when schools are situated in city centers or near high-emission sources without green infrastructure to mitigate pollution. This is particularly relevant given that indoor pollutant concentrations are often strongly influenced by outdoor levels, with studies indicating that indoor concentrations may reach a substantial fraction of those measured outdoors [16,52].

A major challenge in exposure studies remains to accurately measure direct air pollution exposure. In our project, we used the MX-KIT020 miniXT basic and NE-OAM065 NEMo^®^ outdoor (Ethera, France); however this equipment does not measure black carbon or ultrafine particles, which are important when considering health outcomes as they can enter the bloodstream and spread through the body [53,54].

Despite these limitations, the potential of this study should be acknowledged. The current literature has primarily focused on long-term exposure and its impact on development. Studies determining the acute effects of exposure are limited. The main strengths of our study are the inclusion of healthy children and the measurement of multiple health outcomes, combined with real-time air pollution measurements conducted at the school level, independently of fixed governmental monitoring stations, thereby increasing spatial data resolution both in- and outdoors. Our study can also provide a framework to describe possible challenges in longitudinally measuring and linking exposure and health effects in children.

## 5. Conclusions

PM concentrations showed limited associations with cardiovascular or neurocognitive outcomes in healthy children. Direct indoor PM_2.5_ exposure was associated with reduced microvascular dilatation, whereas attentional performance appeared to improve with direct and recent PM exposure. However, these associations did not persist after the exclusion of the open-air school from the analyses, with the exception of selective attention. In contrast, positive associations were revealed between indoor PM_2.5_ and both RHI and selective attention response time. These findings suggest that the inclusion of an open-air school may have influenced our results. Longitudinal studies with a larger sample size, combined with accurate exposure assessment are needed to further elucidate the potential direct effects of PM exposure and disentangle the influence of (an open-air) school environment on children’s learning and development.

## Figures and Tables

**Table 1:** Baseline demographic and anthropometric characteristics. Participant numbers (N) per school are shown for the total sample, endothelial function (EF), and neurocognitive function (NCF) assessments. Results are presented as mean ± SD values or percentages by school (S1–S7).

	S1	S2	S3	S4	S5	S6	S7
***N***	16	37	20	8	20	11	26
N EF	15	31	19	7	17	10	22
N NCF	16	37	20	0	20	11	26
Age (years)	9.9 ± 0.6	10.4 ± 0.4	10.2 ± 0.3	10.2 ± 0.7	10.2 ± 0.4	9.9 ± 0.7	10.6 ± 0.5
Boys (%)	56.2	51.4	45	87.5	55	63.6	57.7
Height (cm)	141.1 ± 8	147.2 ± 7.3	143.0 ± 6.2	147.9 ± 4.4	147.2 ± 5.8	142.0 ± 7.5	146.5 ± 6.5
Weight (kg)	32.9 ± 3.7	38.8 ± 8.9	35 ± 7.6	37.3 ± 9.7	37.5 ± 7.1	36.9 ± 10.5	36.5 ± 6.5
BMI (kg/m^2^)	16.3 ± 1.4	17.8 ± 2.6	16.4 ± 2.9	16.8 ± 3.9	16.4 ± 1.4	17.9 ± 3.5	16.5 ± 2

**Table 2:** Blood pressure, endothelial function, and neurocognitive test results per visit. Mean ± SD or median (Q1–Q3) are presented per visit. P_overall_: *p*-value obtained from linear mixed-effects model, with correction for age at baseline, gender, and BMI-z. For endothelial function, room temperature was added as fixed effect. P_V2-V1_, P_V3-V1_, P_V3-V2_: *p*-values for post hoc comparisons based on the model. If P_overall_ > 0.05, no post hoc comparisons were performed. V_1_, V_2_, V_3_ = Visit 1, 2, 3. SBP = systolic blood pressure; DBP = diastolic blood pressure; RHI = reactive hyperemia Index; tpr = time-to-peak response; MaxRH = maximal dilatation. RT = reaction time; Acc = accuracy; Stroop task is for selective attention measurement; CPT = continuous performance test for sustained attention measurement.

	Visit 1 October 2022	Visit 2 February 2023	Visit 3 May 2023	P_overall_	P_V2-V1_	P_V3-V1_	P_V3-V2_
**Blood pressure**							
Number of participants	133	132	133				
SBP	110 ± 8	113 ± 8	110 ± 9	<0.001	<0.001	0.85	<0.001
DBD	70 ± 6	72 ± 6	70 ± 7	<0.001	0.028	0.12	<0.001
PP	39 ± 6	42 ± 6	40 ± 6	0.001	<0.001	0.29	0.012
**Endothelial function**							
Number of participants	85	81	98				
RHI	1.27 (1.00–1.58)	1.17 (0.97–1.41)	1.25 (1.00–1.47)	0.16			
MaxRH	1.67 (1.41–1.94)	1.74 (1.38–2.09)	1.46 (1.25–1.75)	0.042	0.63	0.063	0.018
tpr (s)	186 ± 64	189 ± 54	175 ± 58	0.050			
**STROOP test**							
Number of participants	120	97	115				
Stroop RT (ms)	1424 ± 252	1336 ± 188	1276 ± 180	<0.001	<0.001	<0.001	0.046
Stroop Acc (%)	91.7 (87.5–95.8)	94.4 (90.3–97.2)	93.1 (90.3–95.8)	<0.001	<0.001	0.002	0.57
**CPT test**							
Number of participants	121	95	118				
CPT RT (ms)	461 ± 38	457 ± 37	445 ± 33	<0.001	0.039	<0.001	0.007
CPT Acc (%)	96.8 (94.7–98.4)	97.3 (95.7–98.9)	97.9 (95.7–98.9)	<0.001	<0.001	<0.001	0.26

## Data Availability

The datasets generated and analyzed during the current study are available from the corresponding author upon reasonable request.

## Supplementary Materials

The following supporting information can be downloaded at: https://www.mdpi.com/article/10.3390/ijerph23081070/s1, Table S1. Associations between blood pressure parameters and air pollutant concentrations; Table S2. Associations between endothelial function parameters and air pollutant concentrations; Table S3. Associations between neurocognitive parameters and air pollutant concentrations.
