# Supplementary material for: Effects of Recent Air Pollution Exposure on Children’s Neurocognitive Functions and the Possible Link with Endothelial Function
Source: Int J Environ Res Public Health. 2026 Aug 18;23(8):1070. doi: 10.3390/ijerph23081070 (PMC13513395; doi:10.3390/ijerph23081070)
Supplement: Supplementary file 1 [file ijerph-23-01070-s001.zip › ijerph-4402437-supplementary.pdf]

Table S1. Associations between blood pressure parameters and air pollutant concentrations

Linear mixed models were fitted separately for each air pollutant variable and adjusted for visit, age, sex, and BMI z-score. SBP = Systolic blood pressure; DBP = diastolic blood pressure; PP = pulse pressure. Standardized coefficients with standard error (SE) and p-values are presented. The analysis was repeated without the openair school for indoor $PM_{2.5}$*. Med = median; P90 and P95 =* 90th *and* 95th *percentile.*

	DBP		SBP		PP	
	Coeff (se)	P	Coeff (se)	P	Coeff (se)	P
$PM_{2.5}$ indoor						
4h						
Med	-0.18 (0.06)	0.005	-0.15 (0.06)	0.014	-0.04 (0.07)	0.6
P90	-0.13 (0.06)	0.036	-0.09 (0.06)	0.16	0.01 (0.07)	0.83
P95	-0.13 (0.06)	0.037	-0.09 (0.06)	0.18	0.02 (0.07)	0.8
24h						
Med	-0.11 (0.08)	0.19	-0.05 (0.08)	0.57	0.04 (0.09)	0.6
P90	-0.09 (0.08)	0.25	-0.03 (0.08)	0.73	0.06 (0.08)	0.49
P95	-0.09 (0.08)	0.28	-0.02 (0.08)	0.8	0.06 (0.08)	0.46
1w						
Med	-0.08 (0.07)	0.25	-0.02 (0.07)	0.79	0.05 (0.08)	0.47
P90	-0.02 (0.07)	0.75	0.03 (0.07)	0.63	0.07 (0.07)	0.31
P95	-0.04 (0.07)	0.57	0.03 (0.07)	0.66	0.09 (0.07)	0.24
PM_1 outdoor						
4h						
Med	-0.11 (0.06)	0.077	-0.09 (0.06)	0.15	-0.02 (0.07)	0.78
P90	-0.09 (0.06)	0.14	-0.09 (0.06)	0.16	-0.04 (0.07)	0.6
P95	-0.08 (0.06)	0.19	-0.08 (0.06)	0.19	-0.03 (0.07)	0.62
24h						
Med	-0.13 (0.08)	0.089	0.01 (0.08)	0.9	0.14 (0.09)	0.13
P90	-0.14 (0.08)	0.082	0.02 (0.09)	0.84	0.15 (0.09)	0.092
P95	-0.15 (0.08)	0.081	0.02 (0.09)	0.83	0.16 (0.09)	0.087
1w						
Med	-0.08 (0.07)	0.26	0.03 (0.07)	0.7	0.11 (0.07)	0.11
P90	-0.03 (0.07)	0.71	0.12 (0.07)	0.091	0.2 (0.07)	0.007
P95	0 (0.07)	0.95	0.15 (0.07)	0.037	0.22 (0.08)	0.005
$PM_{2.5}$ outdoor						
4h						
Med	-0.11 (0.06)	0.08	-0.09 (0.06)	0.15	-0.02 (0.07)	0.78
P90	-0.09 (0.06)	0.14	-0.09 (0.06)	0.16	-0.03 (0.07)	0.61
P95	-0.08 (0.06)	0.18	-0.08 (0.06)	0.19	-0.03 (0.07)	0.62
24h						
Med	-0.13 (0.08)	0.093	0.01 (0.08)	0.89	0.14 (0.09)	0.13
P90	-0.14 (0.08)	0.084	0.02 (0.09)	0.83	0.15 (0.09)	0.092
P95	-0.15 (0.08)	0.082	0.02 (0.09)	0.83	0.16 (0.09)	0.089
1w						
Med	-0.09 (0.07)	0.16	0.02 (0.07)	0.79	0.12 (0.07)	0.099
P90	-0.03 (0.07)	0.64	0.11 (0.07)	0.1	0.2 (0.07)	0.007
P95	0 (0.07)	0.97	0.15 (0.07)	0.044	0.22 (0.08)	0.005
PM_{10} outdoor						
4h						
Med	-0.11 (0.06)	0.078	-0.09 (0.06)	0.15	-0.02 (0.07)	0.78
P90	-0.09 (0.06)	0.14	-0.09 (0.06)	0.16	-0.04 (0.07)	0.6
P95	-0.08 (0.06)	0.18	-0.08 (0.06)	0.19	-0.03 (0.07)	0.62
24h						
Med	-0.13 (0.08)	0.094	0.01 (0.08)	0.88	0.14 (0.09)	0.12
P90	-0.14 (0.08)	0.086	0.02 (0.09)	0.82	0.15 (0.09)	0.089
P95	-0.14 (0.08)	0.086	0.02 (0.09)	0.81	0.16 (0.09)	0.086
1w						
Med	-0.09 (0.07)	0.19	0.03 (0.07)	0.68	0.13 (0.07)	0.075
P90	-0.04 (0.07)	0.56	0.11 (0.07)	0.13	0.2 (0.08)	0.008
P95	-0.02 (0.07)	0.78	0.14 (0.07)	0.064	0.22 (0.08)	0.005
NO_2						
	0.074 (0.11)	0.54	0.088 (0.11)	0.41	0.019 (0.13)	0.89

Without open-air school<br>$PM_{2.5}$ indoor						
4h						
Med	-0.22 (0.06)	< 0.001	-0.29 (0.06)	< 0.001	-0.19 (0.07)	0.006
P90	-0.22 (0.06)	< 0.001	-0.29 (0.06)	< 0.001	-0.19 (0.07)	0.006
P95	-0.22 (0.06)	< 0.001	-0.29 (0.06)	< 0.001	-0.19 (0.07)	0.006
24h						
Med	-0.11 (0.09)	0.22	-0.09 (0.09)	0.3	-0.02 (0.09)	0.8
P90	-0.09 (0.09)	0.29	-0.07 (0.09)	0.41	-0.01 (0.09)	0.89
P95	-0.09 (0.09)	0.32	-0.07 (0.09)	0.44	-0.01 (0.1)	0.88
1w						
Med	-0.08 (0.08)	0.31	-0.07 (0.08)	0.34	-0.03 (0.08)	0.7
P90	-0.07 (0.07)	0.31	-0.04 (0.07)	0.55	0.01 (0.08)	0.9
P95	-0.06 (0.07)	0.37	-0.04 (0.07)	0.58	0.01 (0.08)	0.94

Table S2. Associations between endothelial function parameters and air pollutant concentrations
Linear mixed models were fitted separately for each air pollutant variable and adjusted for visit, age, sex, BMI z-score, and temperature. RHI = Reactive Hyperemic Index; Max RH = maximal dilatation; tpr = time-to-peak response. Standardized coefficients with standard error (SE) and p-values are presented. The analysis was repeated without the open-air school for indoor $PM_{2.5}$. Med = median. P90 and P95 = 90th and 95th percentile.

	RHI (log)		Max RH (log)		Time to max	
	Coeff (se)	P	Coeff (se)	P	Coeff (se)	P
$PM_{2.5}$ indoor						
4h						
Med	0.13 (0.09)	0.16	-0.15 (0.08)	0.067	-0.12 (0.09)	0.2
P90	0.05 (0.09)	0.59	-0.16 (0.08)	0.047	-0.12 (0.09)	0.18
P95	0.03 (0.09)	0.71	-0.16 (0.08)	0.045	-0.12 (0.09)	0.18
24h						
Med	0.08 (0.1)	0.45	-0.07 (0.1)	0.48	-0.18 (0.1)	0.083
P90	0.05 (0.1)	0.65	-0.1 (0.1)	0.32	-0.18 (0.1)	0.074
P95	0.04 (0.11)	0.7	-0.12 (0.1)	0.22	-0.18 (0.1)	0.081
1w						
Med	-0.04 (0.09)	0.63	-0.09 (0.08)	0.28	-0.01 (0.09)	0.95
P90	-0.07 (0.09)	0.43	-0.02 (0.08)	0.8	0.09 (0.09)	0.31
P95	-0.08 (0.09)	0.38	-0.05 (0.08)	0.56	0.06 (0.09)	0.51
PM_1 outdoor						
4h						
Med	0.05 (0.1)	0.64	-0.05 (0.09)	0.56	0.1 (0.1)	0.3
P90	0.04 (0.1)	0.66	-0.04 (0.09)	0.63	0.12 (0.1)	0.19
P95	0.04 (0.1)	0.69	-0.04 (0.08)	0.64	0.12 (0.09)	0.19
24h						
Med	-0.06 (0.12)	0.63	0.19 (0.1)	0.07	0.03 (0.11)	0.75
P90	-0.11 (0.12)	0.39	0.16 (0.11)	0.16	0.04 (0.11)	0.72
P95	-0.11 (0.12)	0.36	0.15 (0.11)	0.18	0.05 (0.12)	0.69
1w						
Med	0.04 (0.09)	0.67	0.09 (0.08)	0.27	0.11 (0.09)	0.2
P90	-0.12 (0.09)	0.19	0.04 (0.08)	0.6	0.04 (0.09)	0.64
P95	-0.15 (0.1)	0.13	0.01 (0.09)	0.87	0.02 (0.1)	0.88
$PM_{2.5}$ outdoor						

4h						
Med	0.05 (0.1)	0.63	-0.05 (0.09)	0.57	0.1 (0.1)	0.3
P90	0.04 (0.1)	0.65	-0.04 (0.09)	0.64	0.12 (0.1)	0.19
P95	0.04 (0.1)	0.69	-0.04 (0.08)	0.64	0.12 (0.09)	0.2
24h						
Med	-0.06 (0.12)	0.64	0.19 (0.1)	0.067	0.03 (0.11)	0.77
P90	-0.1 (0.12)	0.4	0.16 (0.11)	0.14	0.04 (0.12)	0.71
P95	-0.11 (0.12)	0.37	0.16 (0.11)	0.15	0.05 (0.12)	0.66
1w						
Med	0.03 (0.09)	0.71	0.09 (0.08)	0.23	0.11 (0.09)	0.19
P90	-0.12 (0.09)	0.21	0.05 (0.08)	0.55	0.05 (0.09)	0.6
P95	-0.15 (0.1)	0.13	0.02 (0.09)	0.85	0.02 (0.1)	0.85
PM_{10} outdoor						
4h						
Med	0.05 (0.1)	0.63	-0.05 (0.09)	0.57	0.11 (0.1)	0.3
P90	0.04 (0.1)	0.65	-0.04 (0.09)	0.64	0.12 (0.1)	0.19
P95	0.04 (0.1)	0.69	-0.04 (0.08)	0.64	0.12 (0.09)	0.19
24h						
Med	-0.06 (0.12)	0.62	0.19 (0.1)	0.073	0.03 (0.11)	0.78
P90	-0.1 (0.12)	0.41	0.16 (0.11)	0.15	0.04 (0.12)	0.72
P95	-0.11 (0.12)	0.38	0.15 (0.11)	0.17	0.05 (0.12)	0.69
1w						
Med	0.02 (0.09)	0.78	0.1 (0.08)	0.19	0.1 (0.09)	0.27
P90	-0.13 (0.1)	0.18	0.02 (0.09)	0.83	0.04 (0.1)	0.68
P95	-0.15 (0.1)	0.12	0.01 (0.09)	0.87	0.03 (0.1)	0.79
NO_2						
	-0.049 (0.16)	0.76	-0.15 (0.17)	0.42	0.034 (0.14)	0.81

Without open-air school<br>$PM_{2.5}$ indoor						
4h						
Med	0.23 (0.09)	0.007	-0.07 (0.08)	0.39	0 (0.09)	0.98
P90	0.21 (0.09)	0.023	-0.1 (0.08)	0.23	0.03 (0.09)	0.74
P95	0.17 (0.09)	0.052	-0.1 (0.08)	0.21	0.03 (0.09)	0.71
24h						
Med	0.06 (0.11)	0.55	0 (0.1)	0.97	-0.12 (0.11)	0.26
P90	0.03 (0.11)	0.77	-0.03 (0.1)	0.78	-0.12 (0.11)	0.25
P95	0.03 (0.11)	0.82	-0.05 (0.1)	0.65	-0.12 (0.11)	0.29
1w						
Med	-0.03 (0.1)	0.77	-0.09 (0.09)	0.32	-0.03 (0.09)	0.76
P90	-0.06 (0.09)	0.52	-0.06 (0.08)	0.46	-0.03 (0.09)	0.76
P95	-0.08 (0.09)	0.4	-0.07 (0.08)	0.39	-0.03 (0.09)	0.78

Table S3. Associations between neurocognitive parameters and air pollutant concentrations

Linear mixed models were fitted separately for each air pollutant variable and adjusted for visit, age, sex, and BMI z-score. RT = Response time; ACC = accuracy. Stroop task is for selective attention measurement. CPT= continuous performance test for sustained attention measurement. Standardized coefficients with standard error (SE) and p-values are presented. The analysis was repeated without the open-air school for indoor $PM_{2.5}$. Med = median. P90 and P95 = 90th and 95th percentile.

	RT Stroop		ACC Stroop		RT CPT		ACC CPT	
	Coeff (se)	P	Coeff (se)	P	Coeff (se)	P	Coeff (se)	P
$PM_{2.5}$ indoor								
4h								
Med	-0.06 (0.07)	0.43	0.16 (0.07)	0.034	-0.08 (0.07)	0.3	0.12 (0.08)	0.12
P90	-0.09 (0.07)	0.22	0.16 (0.08)	0.031	-0.09 (0.08)	0.22	0.12 (0.08)	0.12
P95	-0.09 (0.07)	0.24	0.16 (0.08)	0.034	-0.09 (0.08)	0.22	0.12 (0.08)	0.13
24h								
Med	0.13 (0.09)	0.13	0.23 (0.09)	0.012	0.04 (0.09)	0.69	0.16 (0.1)	0.099
P90	0.11 (0.08)	0.2	0.23 (0.09)	0.011	0 (0.09)	0.96	0.17 (0.09)	0.067
P95	0.09 (0.08)	0.29	0.23 (0.09)	0.013	-0.01 (0.09)	0.89	0.18 (0.09)	0.059
1w								
Med	0.12 (0.08)	0.14	0.14 (0.08)	0.088	-0.03 (0.09)	0.71	0.17 (0.09)	0.058
P90	0.14 (0.07)	0.061	0.05 (0.08)	0.51	-0.03 (0.08)	0.68	0.15 (0.08)	0.055
P95	0.1 (0.08)	0.23	0.1 (0.08)	0.23	-0.06 (0.08)	0.43	0.18 (0.08)	0.037
PM_1 outdoor								
4h								
Med	-0.13 (0.07)	0.065	0.07 (0.07)	0.36	-0.08 (0.07)	0.3	0.07 (0.07)	0.37
P90	-0.11 (0.06)	0.077	0.01 (0.07)	0.84	-0.05 (0.07)	0.43	0.05 (0.07)	0.51
P95	-0.11 (0.06)	0.083	0.01 (0.07)	0.94	-0.05 (0.07)	0.46	0.04 (0.07)	0.54
24h								
Med	0.12 (0.08)	0.15	0.05 (0.1)	0.62	-0.02 (0.09)	0.87	-0.04 (0.09)	0.65
P90	0.09 (0.09)	0.32	0.06 (0.1)	0.57	-0.05 (0.09)	0.62	-0.03 (0.1)	0.79
P95	0.08 (0.09)	0.37	0.05 (0.1)	0.59	-0.05 (0.1)	0.59	-0.02 (0.1)	0.82
1w								
Med	0.05 (0.08)	0.51	0.02 (0.08)	0.84	-0.03 (0.08)	0.71	0.03 (0.08)	0.72
P90	0.11 (0.08)	0.18	0.09 (0.08)	0.3	-0.05 (0.08)	0.55	0.03 (0.08)	0.68
P95	0.11 (0.08)	0.16	0.13 (0.08)	0.12	-0.06 (0.08)	0.48	0.06 (0.08)	0.49
$PM_{2.5}$ outdoor								
4h								
Med	-0.13 (0.07)	0.066	0.07 (0.07)	0.37	-0.07 (0.07)	0.3	0.07 (0.07)	0.37
P90	-0.11 (0.06)	0.078	0.01 (0.07)	0.84	-0.05 (0.07)	0.43	0.05 (0.07)	0.51
P95	-0.11 (0.06)	0.084	0.01 (0.07)	0.93	-0.05 (0.07)	0.46	0.04 (0.07)	0.54
24h								
Med	0.12 (0.08)	0.15	0.05 (0.1)	0.59	-0.02 (0.09)	0.86	-0.04 (0.09)	0.67
P90	0.09 (0.09)	0.31	0.05 (0.1)	0.59	-0.05 (0.1)	0.63	-0.03 (0.1)	0.78
P95	0.09 (0.09)	0.32	0.05 (0.1)	0.66	-0.05 (0.1)	0.61	-0.03 (0.1)	0.76
1w								
Med	0.06 (0.08)	0.47	0.04 (0.08)	0.65	-0.05 (0.08)	0.57	0.03 (0.08)	0.69
P90	0.11 (0.08)	0.17	0.08 (0.08)	0.31	-0.05 (0.08)	0.54	0.03 (0.08)	0.71
P95	0.11 (0.08)	0.16	0.13 (0.08)	0.11	-0.06 (0.08)	0.45	0.06 (0.08)	0.49
PM_{10} outdoor								
4h								

Med	-0.13 (0.07)	0.066	0.07 (0.07)	0.36	-0.08 (0.07)	0.3	0.07 (0.07)	0.36
P90	-0.11 (0.06)	0.078	0.01 (0.07)	0.85	-0.05 (0.07)	0.43	0.05 (0.07)	0.51
P95	-0.11 (0.06)	0.083	0.01 (0.07)	0.93	-0.05 (0.07)	0.46	0.04 (0.07)	0.54
24h								
Med	0.12 (0.08)	0.16	0.05 (0.1)	0.57	-0.02 (0.09)	0.84	-0.04 (0.09)	0.69
P90	0.09 (0.09)	0.34	0.06 (0.1)	0.56	-0.05 (0.09)	0.62	-0.02 (0.1)	0.81
P95	0.08 (0.09)	0.38	0.06 (0.1)	0.58	-0.05 (0.1)	0.58	-0.02 (0.1)	0.83
1w								
Med	0.07 (0.08)	0.33	0.03 (0.08)	0.68	-0.03 (0.08)	0.68	0.01 (0.08)	0.86
P90	0.1 (0.08)	0.21	0.12 (0.08)	0.16	-0.06 (0.08)	0.49	0.06 (0.09)	0.51
P95	0.1 (0.08)	0.21	0.14 (0.08)	0.089	-0.07 (0.08)	0.37	0.06 (0.08)	0.45
NO_2								
	-0.2 (0.14)	0.17	-0.043 (0.12)	0.74	0.011 (0.15)	0.94	-0.0068 (0.13)	0.96

Without open-air school<br>$PM_{2.5}$ indoor								
4h								
Med	0.06 (0.06)	0.37	0.06 (0.07)	0.45	0.01 (0.07)	0.89	0.07 (0.07)	0.32
P90	0.06 (0.06)	0.34	0.05 (0.07)	0.5	0.01 (0.07)	0.85	0.08 (0.07)	0.24
P95	0.07 (0.06)	0.24	0.04 (0.07)	0.56	0.02 (0.07)	0.81	0.08 (0.07)	0.24
24h								
Med	0.19 (0.09)	0.043	0.21 (0.1)	0.047	0.08 (0.11)	0.44	0.13 (0.11)	0.24
P90	0.18 (0.09)	0.048	0.2 (0.1)	0.051	0.04 (0.1)	0.68	0.15 (0.11)	0.18
P95	0.18 (0.09)	0.059	0.19 (0.1)	0.063	0.04 (0.11)	0.68	0.15 (0.11)	0.17
1w								
Med	0.25 (0.08)	0.003	0.09 (0.09)	0.31	0.04 (0.09)	0.64	0.11 (0.09)	0.24
P90	0.23 (0.07)	0.002	0.11 (0.08)	0.18	0 (0.08)	0.97	0.11 (0.08)	0.21
P95	0.22 (0.07)	0.003	0.11 (0.08)	0.2	0 (0.08)	0.95	0.11 (0.08)	0.17
